# Impact of land use land cover changes on ecosystem service value – A case study of Guangdong, Hong Kong, and Macao in South China

**DOI:** 10.1371/journal.pone.0231259

**Published:** 2020-04-08

**Authors:** Sarah Hasan, Wenzhong Shi, Xiaolin Zhu

**Affiliations:** Department of Land Surveying and Geo-informatics, The Hong Kong Polytechnic University, Hong Kong, Hong Kong; Gebze Teknik Universitesi, TURKEY

## Abstract

The rapid increase in anthropogenic activities, socioeconomic development, and land use land cover (LULC) changes since the opening of economic reforms (1978), have changed the ecosystem service value (ESV) in Guangdong, Hong Kong, and Macao (GKHM) region located in South China. This leads to the requirement of a significant tailored analysis of ecosystem services regarding incisive and relevant planning to ensure sustainability at regional level. This study focuses on the use of Landsat satellite imagery to quantify the precise impact of LULC changes on the ecosystem services in GHKM over the past three decades (1986–2017). The most renowned established unit value transfer method has been employed to calculate the ESV. The results show that the total ecosystem service value in GHKM has decreased from 680.23 billion CNY in 1986 to 668.45 billion CNY in 2017, mainly due to the decrease in farmland and fishponds. This overall decrease concealed the more dynamic and complex nature of the individual ESV. The most significant decrease took place in the values of water supply (-22.20 billion CNY, -14.72%), waste treatment (-20.77 billion CNY, −14.63%), and food production (-7.96 billion CNY, −33.18%). On the other hand, the value of fertile soil formation and retention (6.28 billion CNY, +7.26%) and recreation and culture (5.09 billion CNY, +12.91%) increased. Furthermore, total ESV and ESV per capita decreased significantly with the continuous increase in total gross domestic product (GDP) and GDP per capita. A substantial negative correlation exists between farmland ESV and GDP indicating human encroachment into a natural and semi natural ecosystems. The results suggest that in the rapidly urbanizing region, the protection of farmland and to control the intrusion of urban areas has marked an important societal demand and a challenge to the local government. This required a pressing need for smart LULC planning and to improve policies and regulation to guarantee ecosystem service sustainability for acceptable life quality in the study area and other fast expanding urban areas in China.

## Introduction

Ecosystem services (ES) can be described as the condition and processes through which natural ecosystems, and the species that comprise them, sustain and fulfill human well-being [1–3]. Ecosystem services can be considered as the goods and services that benefit human life both directly and indirectly [4,5]. These services include supporting services, regulating services, provisioning services, and cultural services. These services incorporate benefits to the society [5–7]. In connection to rapid economic development, intense human activities and urbanization in the fastest burgeoning developing countries has placed pressure in the deterioration of key ES [8–10]. Thus, the endowment of ES, its structure, and functions greatly influenced by changes in patterns, practices, and intensity of land use land cover (LULC) [10–14]. Such changes in land cover have put both ecosystems and humans at risk and are expected to continue to increase in the future [5,15,16]. Therefore, increasing imbalance provision of ecosystems under the rapidly growing urbanization and development have become a focus of concern [7]. Such situations are more pronounced in developing countries such as China.

Since China initiated the opening of economic reform and policy in 1978, socioeconomic development and adaptation of several land use policies have driven significant changes in LULC with increasing speed, breath, and depth [17]. These changes have resulted in the urban expansion, loss of farmland [16,18], ecological damage [7], and horticultural development without proper planning and management of prevailing land resources [15]. China’s total urban population has increased from 11.80% in 1950 to 58.52% in 2017, which is predicted to reach 76.10% by the end of 2050 [19]. The increasing human populace and socioeconomic development have confronted genuine difficulties in ecological land, various ecosystem services value, and food security both in space and time [5,16]. Thus, knowledge of economic valuation, analysis, and quantification of the effect of ongoing development on ES are necessary for policy decision makers in both the exploration of the means to achieve socioeconomic and ecological sustainable development [20–22]. Therefore, in recent year, ecosystem services have started gaining importance in order to reveal the coevolution process of both nature and human [5].

Several studies have been performed to monitor the impact of LULC changes on the structure and functions of ESV in numerous regions of China [4,10,18,23–33] and around the world [15,25,28,29,34–45]. Due to LULC changes and urbanization, most of these studies showed moderate to significant decrease in ESV [15,16,28,29,41,43] while others found almost no change [46]. Some studies have also revealed an increase in ESV [47–50]. Such variations in the services value are of the following reasons. Firstly, in terms of fast urbanization and industrialization, several changes in LULC occur concurrently, as a result of not only limited to urban sprawl but also include various contending demands. These include reforestation and protection, natural indemnity, infrastructure development, comfort, and tourism and recreation [51,52]. Secondly, there is a very close relationship between ESV types and LULC utilization [20,53]. Given their profound implication and distinct nature and characteristics, the capability to summarize and apply the results of these case studies to other regions is limited. Thus, indicating the importance of studying the impact of LULC changes on ESV, which is essential to inform policy and decision-makers for sustainable planning and management and safe ecological system.

Several methods exist to enable the quantification of the global terrestrial ecosystem services value but the method most commonly used is the “benefit transfer” developed by Costanza’s et al. (1997) [2]. They classified the world ecosystems into 16 types and 17 subtypes as their services functions. Their results, however, have been seriously condemned when applied to China. For example, bias in some cases such as underestimated farmland ESV and overestimated wetland ESV [35]. Their derived ESV mirrored the economic level of developed countries (e.g., United States and European countries) instead of developing countries such as China [18,25,54]. For Chinese terrestrial ecosystem services Xie et al. (2003) developed the equivalent per-unit-area following the same methodology proposed by Costanza et al. (1997) [2]. They extracted the equivalent weight factor via a survey of 200 Chinese ecologists. Combined with land use data, equivalent per-unit-area values were widely used in a different regions of China to calculate ESV [4,10,35,37,55]. Using this method, LULC can act as a proxy by coordinating the land cover type proportional to the biomes. The later then assign the economic values centered on a standard, adjusted locally, and value of coefficients set. This method provide a type of multi-criteria technique, enabling the integration of diverse distinctive measurements into a solitary money related unit. Furthermore, this approach provides repeatable and comparable results, an assessment of change with time, and crosswise over a heterogeneous urbanization perspective. Hence, it gives a constant mode to enhance knowledge with time through different case studies [10,20,25].

The present study focus on Guangdong, Hong Kong, and Macao (GHKM) located in South China. Since 1978, economic development and urban expansion in GHKM has caused this region to become one of the fastest developing regions in the world. This has resulted in harsh natural conditions, overconsumption, and deterioration of provisioning services from nature and put both ecosystems and well-being at risk [5,10,20]. The Chinese government has initiated different measures to improve the deteriorated ecological environment, by means of such as an increase in forest cover and to protect high productive cropland. The imbalance provision of ES is the main restricted factor both in social and economic sustainable development in the GHKM [7]. Therefore, no such studies have been conducted in GHKM that provides a comprehensive understanding and estimating the impact of such changes in land cover and policies on the ES. Hence, the objective of this study are as follows: to evaluate and quantify the effect of LULC changes on ESV in GHKM from 1986 to 2017, to assign the specific coefficient of ESV to each land use category using the established unit-value transfer method, and to scrutinize the impact of LULC changes on ESV. The coefficient of sensitivity is then assessed to estimate the uncertainty in the value coefficient. On the bases of the results, this study also aims to provide information useful to urban planners and decision-makers for the regional coordinate and sustainable development.

## Methodology

### Study area

**Fig 1:**
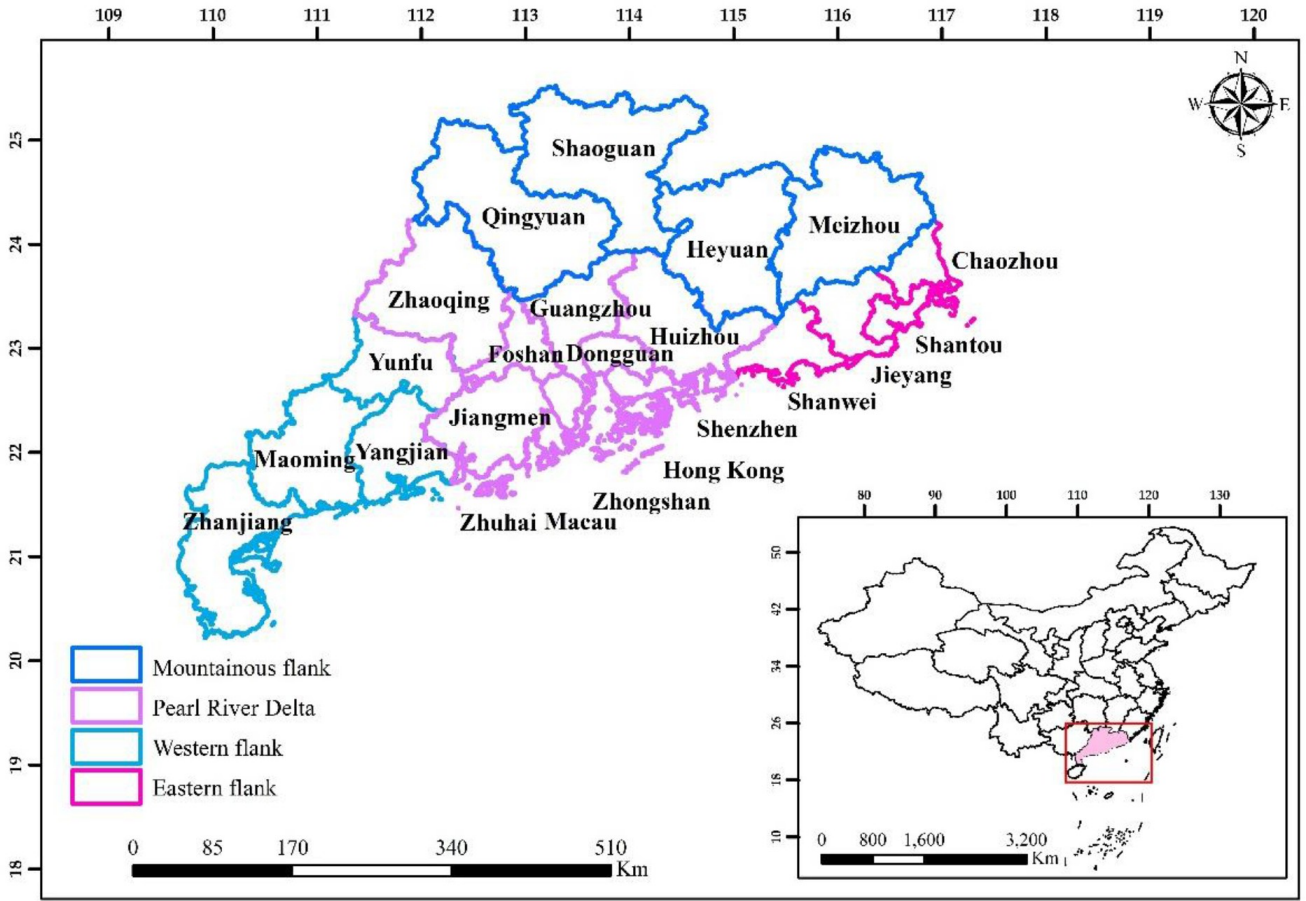
The geographical location of Guangdong, Hong Kong, and Macao, South China. The map were created using software ArcGIS 10.1 [61].

GHKM, a tropical and subtropical region geographically located in the southern part of China (Fig 1). GHKM is one of the largest political, economical, and cultural centers of southern China. Its total area coverage is about 196,342 km^2^ with a total population of 9164.90 (10,000 persons) in 2017. It comprises of 23 cities, are divided into four groups, namely mountainous region, Pearl River Delta (PRD), eastern side, and western side according to their geographical location. It shares borders with Guangxi province in the west, Hunan and Jiangxi provinces in the north, Fujian province in the east, and the South China Sea in the south [56,57]. It has a tropical and subtropical monsoon rainy season, beginning in April and ending in September. Its climate is humid with a yearly mean temperature of 22°C and a yearly mean rainfall of 1500–2000 mm. Its topography shows various and complex terrain forms including mountains, hills, plateaus, and plains [56–59]. Over the past three decades, the GDP of the GHKM has increased from 66.75 (billion CNY) in 1986 to 7951.21 (billion CNY) in 2017 [60].

### Acquisition of data and land use land cover classification

LULC data play a pivotal role to evaluate the ESV and the availability of historical LULC data provides an adequate ground to analyze changes in ESV [62]. The LULC data for the GHKM have been produced in our previous study [60] which is based on the classification of multi-temporal Landsat images (TM/ETM+/OLI) at 30m resolution for the years 1986, 1989, 1994, 2000, 2005, 2010, and 2017. Each LULC map comprises of the seven classes according to the China National Standard Land Use Classification System (Table 1). The overall accuracy of the classified LULC maps was about 91% and Kappa 0.88 [57]. To detect LULC changes, a cross-tabulation detection method was used to quantify the transitions. The LULC changes, related to seven images, were also mapped and graphed [57]. The data was then used to estimate changes in various ESV and spatial analyses.

**Table 1:** Description of land use land cover classes.

Class	Description
Forest	Forest and Tree cover
Grassland	Natural Shrubs and grassland, constructed grassland, and meadows
Water	Natural water bodies, oceans, lakes, rivers, streams, and reservoir. Water bodies not used for intensive aquaculture
Fishponds	Water bodies used for intensive aquaculture. Dike pond, including mulberry.
Built-up	Land used for man-made structures
Bareland	Sand, rocks, Bare soil, landfill sites, and active excavation areas
Farmland	Land used for farming, cropland, and orchards

### Assigning Ecosystem Service Value (ESV)

In this study, we used the ES classification which is based on nine ecosystem services (Table 2) proposed by Xie et al. (2003). By tailoring the localized average natural grain yield, the equivalent weight factor, as shown in Table 2, can be applied to different regions of China. As a benchmark, the economic value of average natural grain production of farmland per year was set at 1.0 [10,25,31,39,41,46]. Based on this factor, all other coefficients were adjusted accordingly. Xie et al. (2003) proposed that in general, the natural food production should be 1/7 of the actual food production [25,63]. From 1986 to 2017, GHKM’s average actual grain production was 5529.76 kg/ ha and the average grain price in 2017 was 2.65 CNY/kg. Thus, the ESV of one equivalent weight factor for GHKM is 2093.41 CNY ha^-1^ (5529.76*2.65/7).

**Table 2:** Equivalent weighting factor per hectare ESV of Chinese terrestrial ecosystems (Xie et al. (2003)).

	Forest	Grassland	Water	Fishponds	Built-up	Bareland	Farmland
**Food**	0.1	0.3	0.1	0.3	0.01	0.01	1
**Raw material**	2.6	0.05	0.01	0.07	0	0	0.1
**Gas regulation**	3.5	0.8	0	1.8	-2.42	0	0.5
**Climate regulation**	2.7	0.9	0.46	17.1	0	0	0.89
**Water supply**	3.2	0.8	20.4	15.5	-7.51	0.03	0.6
**Waste treatment**	1.31	1.31	18.2	18.18	-2.46	0.01	1.64
**Soil formation and retention**	3.9	1.95	0.01	1.71	0.02	0.02	1.46
**Biodiversity protection**	3.26	1.09	2.49	2.5	0.34	0.34	0.71
**Recreation and culture**	1.28	0.04	4.34	5.55	0.01	0.01	0.01
**Total**	21.85	7.24	46.01	62.71	-12.01	0.42	6.91

On the basis of the linkage between LULC types and biome types, the ESV per unit area of each LULC class in GHKM was assigned (Table 2). Specifically, LULC types “forest”, “grassland”, “water”, “fishponds”, “built-up”, “bare land”, and “farmland” equal to biome types “woodland”, “grassland”, “water body”, “wetland”, “construction land”, “unused land”, and “cropland”, respectively. For built-up, the coefficient value proposed by following Dong et al. (2007) [64] and Deng (2012) [65] was considered. In this study, although the biomes used as proxies for each type of LULC do not perfectly match in each case however, they are related [36]. Their use has been proven feasible in other case studies [18,25,29,41].

### Ecosystem service value calculation

By using Eq (1), Eq (2), and Eq (3) the ecosystem service value, ecosystem function, and total ESV for each thematic class was determined after evaluating the ESV per unit area for each land cover class [4,41,66,67].

$${ESV}_{k}=\sum_{f}A_{k}*{VC}_{kf}$$ (1)

$${ESV}_{f}=\sum_{k}A_{k}*{VC}_{kf}$$ (2)

$$ESV=\sum_{k}\sum_{f}A_{k}*{VC}_{kf}$$ (3)

Where, *ESV_k_* the ESV for LULC class “k”, *ESV_f_* represents the value of ecosystem function type “f”, and *ESV* represents the total ESV respectively. *A_k_* represents the area for LULC class “k” and *VC_kf_* represents the value coefficient (CNY/ha/a) for LULC class “k” and ecosystem function type “f” [4,41,66].

### Sensitivity analysis

Since the biomes used as proxies do not perfectly match the LULC class (as mention above in section assigning ecosystem service value (ESV)) and there exist uncertainties in the coefficient values, sensitivity analysis is needed to determine the dependence level of the change of the ESV upon the coefficient values. Therefore, the standard economic elasticity concept was used to calculate the coefficient of sensitivity (CS) as follows:

$$CS=\left|\frac{({ESV}_{j}-{ESV}_{i})/{ESV}_{i}}{({VC}_{jk}-{VC}_{ik})/{VC}_{i}}\right|$$ (4)

The percentage change in the ESV calculated resulting from ± 50% change in the coefficient value and LULC class ‘*k’*. “i” and “j” indicate the respective initial and adjusted values. If CS > 1, the estimated ESV is elastic, relative to that coefficient, whereas if CS < 1 than the estimated ESV is considered to be inelastic. The more prominent the corresponding change in the ESV with respect to a relative change in the coefficient value, the more serious is the utilization of a precise ecosystem value coefficient. However, in previous studies, the sensitivity analysis has been widely used [4,16,20,25,36,41,54,67,68].

## Results

### Land use land cover change

**Fig 2:**
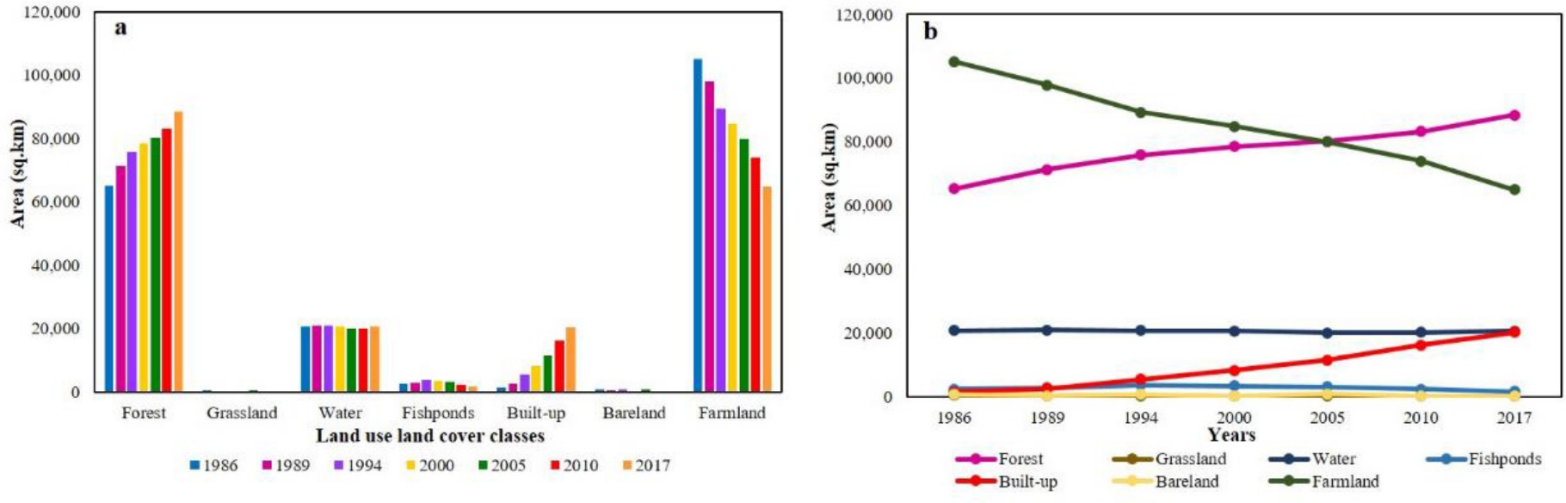
Land use land cover area distribution of different classes (a) and change trend (b) in Guangdong, Hong Kong, and Macao over the study period (1986–2017).

GHKM LULC changed substantially between 1986 and 2017 (Fig 2). Farmland had the greatest decline in the area among the seven LULC classes (-40191.84 km^2^, -38.23%), followed by fishponds (-788.61 km^2^, -32%) and water (-152.22 km^2^, -0.73%). On the other hand, forest exhibited the largest increase (23126.88 km^2^, 35.40%), followed by built-up land (18753.44 km^2^, 1260.02%). As compared to other thematic classes, the built-up area increases with the highest annual growth rate i.e., 8.41% (Table 3). The estimated size of both water and fishponds were relatively small but they both play a vital role in ES and often have high service value. Their cumulative area accounts for only 11% of GHKM’s total area, which even seemed to declines during socioeconomic development and urbanization. The major transformation observed were farmland into built-up land and forest whereas, fishponds into built-up land (S1 Table). Thus, farmland and fishponds are the primary contributors to the new built-up areas. The transformation among different LULC classes certainly affects ecosystems structures and functions as well as variation in the total ESV. Therefore, estimating changes in the ESV in response to LULC changes are described in the below sections.

**Table 3:** Annual rate of change.

Land use type	1986–1989	1989–1994	1994–2000	2000–2005	2005–2010	2010–2017	1986–2017
**Forest**	2.95	1.23	0.58	0.42	0.75	0.86	0.98
**Grassland**	-19.02	6.51	-8.21	11.96	-21.00	5.10	-2.69
**Water**	0.19	-0.02	-0.19	-0.50	0.06	0.30	-0.02
**Fishponds**	6.08	4.94	-1.36	-2.02	-4.94	-5.51	-1.24
**Built-up**	19.15	14.37	7.21	6.26	6.93	3.17	8.41
**Bareland**	-21.40	13.36	-17.08	24.64	-29.47	3.45	-3.22
**Farmland**	-2.38	-1.83	-0.86	-1.16	-1.60	-1.85	-1.55

### Variations in ecosystem service value

In this study, based on the Eqs (1)–(3) the ESV of each land cover class and total ESV of the GHKM for the years 1986, 1989, 1994, 2000, 2005, 2010, and 2017 were calculated using the modified value coefficients (Table 4) and the area of each LULC (Fig 2). According to the results, shown in Table 5, it can be indicated that the general ESV trend is characterized by a variable change process. During the study period, in GHKM’s total ESV surged from 680.23 billion CNY in 1986 to 713.68 billion CNY in 1994, then declined to 668.45 billion CNY in 2017. In the first eight years (1986–1994), the total value of ESV increased by approximately 33.45 billion CNY. The ESV net benefits per hectare was 1703.38 CNY. In the following 23 years (1994–2017), ESV loss was about 45.22 billion CNY, and the net loss of ESV per hectare was 2303.04 CNY. This net gain and loss in ESV are due to the LULC changes during the study period.

**Table 4:** Per unit area ESV of different LULC classes in Guangdong, Hong Kong, and Macao (CNYha^-1^year^-1^).

	Forest	Grassland	Water	Fishponds	Built-up	Bareland	Farmland
**Gas regulation**	7326.94	1674.73	0.00	3768.14	-5066.05	0.00	1046.71
**Climate regulation**	5652.21	1884.07	962.97	35797.31	0.00	0.00	1863.13
**Water supply**	6698.91	1674.73	42705.56	32447.86	-15721.51	62.80	1256.05
**Soil formation and retention**	8164.30	4082.15	20.93	3579.73	41.87	41.87	3056.38
**Waste treatment**	2742.37	2742.37	38100.06	38058.19	-5149.79	20.93	3433.19
**Biodiversity protection**	6824.52	2281.82	5212.59	5233.53	711.76	711.76	1486.32
**Food**	209.34	628.02	209.34	628.02	20.93	20.93	2093.41
**Raw material**	5442.87	104.67	20.93	146.54	0.00	0.00	209.34
**Recreation and culture**	2679.56	83.74	9085.40	11618.43	20.93	20.93	20.93
**Total**	45741.01	15156.29	96317.79	131277.74	-25141.85	879.23	14465.46

**Table 5:** Total ESV for each land use type in Guangdong, Hong Kong, and Macao from 1986 to 2017.

**ESV billion CNY**		**Forest**	**Grassland**	**Water**	**Fishponds**	**Built-up**	**Bareland**	**Farmland**	**Total**
	**1986**	298.50	0.70	200.30	32.34	-3.74	0.07	152.07	680.23
	**1989**	326.18	0.40	201.38	38.59	-6.65	0.03	148.35	708.28
	**1994**	346.90	0.53	201.21	49.46	-13.69	0.07	129.19	713.68
	**2000**	359.15	0.33	199.07	45.64	-21.07	0.02	122.69	705.84
	**2005**	366.72	0.61	194.13	41.38	-28.87	0.08	115.79	689.84
	**2010**	380.64	0.22	194.65	32.18	-40.75	0.02	106.90	673.84
	**2017**	404.28	0.29	198.83	21.99	-50.89	0.02	93.93	668.45
**1986–1989**	billion CNY	27.68	-0.30	1.07	6.25	-2.91	-0.03	-3.72	28.05
	**%**	9.27	-42.53	0.54	19.32	77.71	-47.15	-2.45	4.12
	**%/yr**	3.00	-16.86	0.18	6.06	21.13	-19.15	-0.82	1.36
**1989–1994**	billion CNY	20.72	0.13	-0.17	10.88	-7.04	0.03	-19.15	5.40
	**%**	6.35	32.48	-0.08	28.19	105.81	90.29	-12.91	0.76
	**%/yr**	1.24	5.79	-0.02	5.09	15.53	13.73	-2.73	0.15
**1994–2000**	billion CNY	12.26	-0.20	-2.14	-3.82	-7.39	-0.04	-6.50	-7.84
	**%**	3.53	-38.54	-1.06	-7.73	53.97	-63.09	-5.03	-1.10
	**%/yr**	0.58	-7.79	-0.18	-1.33	7.46	-15.30	-0.86	-0.18
**2000–2005**	billion CNY	7.56	0.28	-4.94	-4.26	-7.80	0.06	-6.90	-16.00
	**%**	2.11	85.51	-2.48	-9.34	36.99	234.30	-5.62	-2.27
	**%/yr**	0.42	13.15	-0.50	-1.94	6.50	27.30	-1.15	-0.46
**2005–2010**	billion CNY	13.92	-0.39	0.51	-9.20	-11.89	-0.06	-8.90	-16.00
	**%**	3.80	-64.10	0.26	-22.23	41.18	-76.72	-7.68	-2.32
	**%/yr**	0.75	-18.53	0.05	-4.90	7.14	-25.29	-1.59	-0.47
**2010–2017**	billion CNY	23.65	0.07	4.19	-10.19	-10.14	0.01	-12.97	-5.39
	**%**	6.21	32.32	2.15	-31.67	24.87	26.71	-12.13	-0.80
	**%/yr**	1.21	5.76	0.43	-7.33	4.54	4.85	-2.55	-0.16
**1986–2017**	billion CNY	105.78	-0.41	-1.47	-10.35	-47.15	-0.04	-58.14	-11.77
	**%**	35.44	-58.77	-0.73	-32.01	1260.02	-63.39	-38.23	-1.73
	**%/yr**	0.98	-2.82	-0.02	-1.24	8.78	-3.19	-1.54	-0.06

A substantial decrease in total ESV (1.73%) between 1986 and 2017 was due to loss of semi-natural land cover types, especially shrinkage in farmland and unprecedented increase in urbanization. However, the loss of farmland was far higher than the loss by urbanization (Table 5). This causes a significant effect in loss of ESV. Though the ESV of other LULCs had increased, such increase was too small to counterbalance the decline. Despite the fact that both water and fishponds covered small areas but they had the highest value coefficients. Therefore, they produced a service value nearly equal to that of the forest. High service value was also produced by farmland due to its large area coverage. The accumulated ESV of forest, water, fishponds, and farmland exceeded 90% of the total value, showing that these land cover classes played a key role in ecosystem services. This is particularly true regarding fishponds whose area was only 0.85–1.9%, yet produced 3–7% of the total ESV. It is assumed that the ESV for bareland and built-up land is much lower due to its low value coefficients.

### Change in ecosystem function

**Fig 3:**
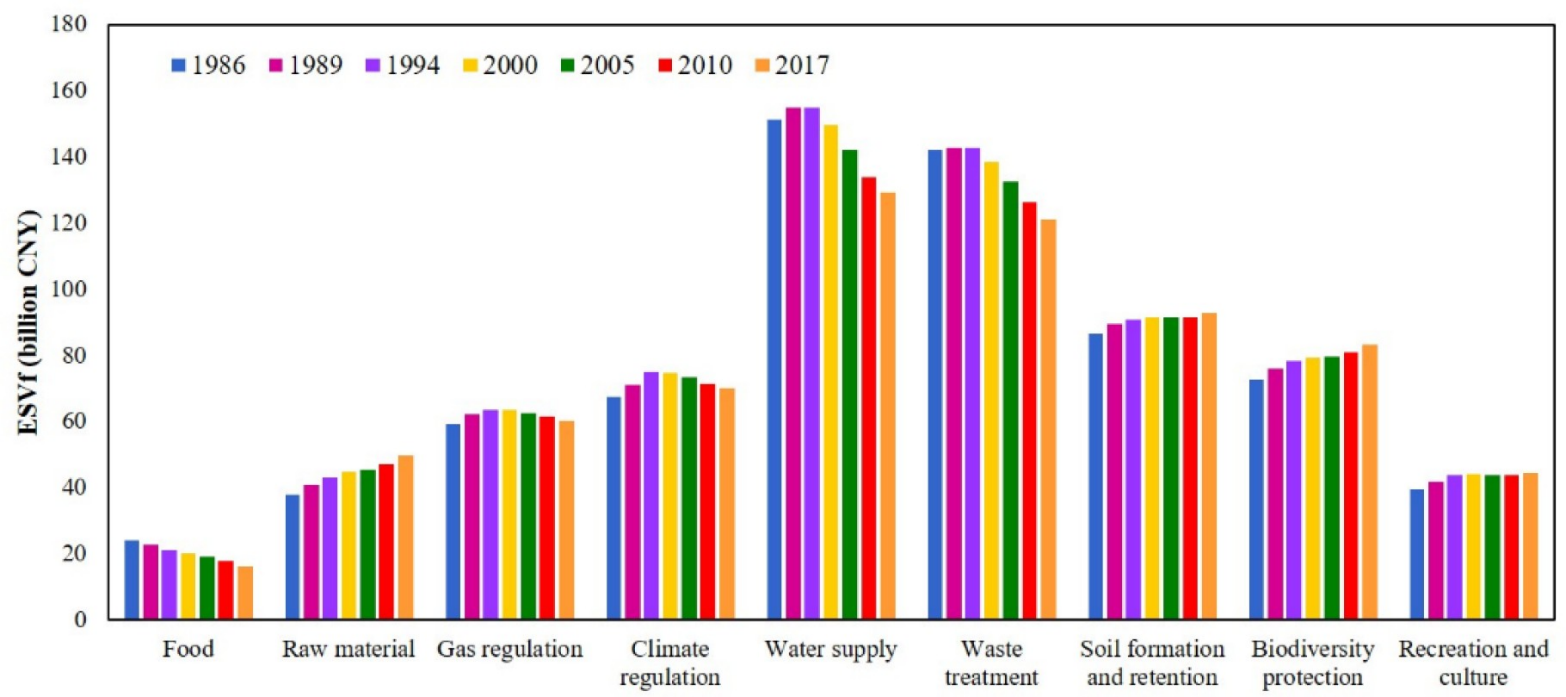
Value of individual ESV in the Guangdong, Hong Kong, and Macao from 1986 to 2017.

The individual ecosystem function (computed using Eq 2) contribution rate to the total ESV are ranked on the basis of their estimated average *ESV_f_* for the years 1986, 1989, 1994, 2000, 2005, 2010, and 2017 (S2 Table). Water supply, waste treatment, soil formation and retention, and biodiversity protection were the most valuable ecosystem services, affecting the total ESV. However, their combined contribution accounted for 65.07%. The highest decline occurred in the water supply value (-22.30 billion CNY, -14.72%) between 1986 and 2017 followed by waste treatment (-20.77 billion CNY, −14.63%) and food production (-7.96 billion CNY, −33.18%). Conversely, soil formation and retention (6.28 billion CNY, +7.26%) and recreation and culture (5.09 billion CNY, +12.91%) have experienced a significant increase in value (Fig 3). Recreation and culture and food production made the least contribution to the ESV, with their accumulated contribution rate was only approximately 9.14%.

**Fig 4:**
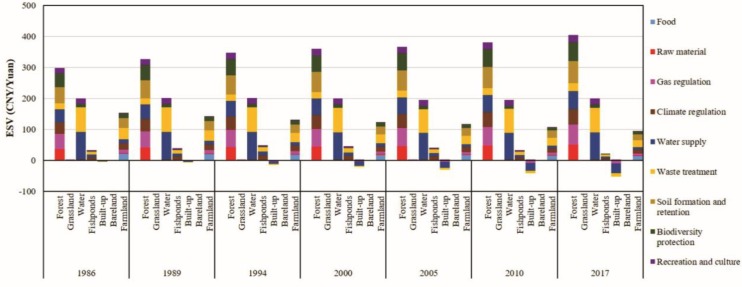
Individual ESV for different land use land cover in the Guangdong, Hong Kong, and Macao from 1986 to 2017.

Due to the large area and the high coefficient value, forest produced the highest ESV among the seven LULC classes i.e., 50% of the total value. It has a significant effect on biodiversity protection, gas regulation, water supply, climate regulation, and soil formation and retention (Fig 4). In 1986 water ESV was 200.30 billion CNY, which decreased by 1.47 billion CNY by 2017 with a robust influence on water supply and waste treatment. Farmland ESV was most affected by LULC changes, decreased by 58.14 billion CNY (38.23%) between 1986 and 2017. This has influenced soil formation and retention, waste treatment, biodiversity protection, and food production. Built-up area, increased by 18,753km^2^ (1260.02%) between 1986 and 2017, produced increasingly negative ESV (47.15 billion CNY), notably through effects on water supply, waste treatment, and gas regulation (Fig 4). However, the increase in the built-up area did not increase the ESV, as its coefficient value was zero, close to zero, and less than zero. This resulted in a rapid reduction in the individual value of ecosystem functions.

### Spatial distribution

**Fig 5:**
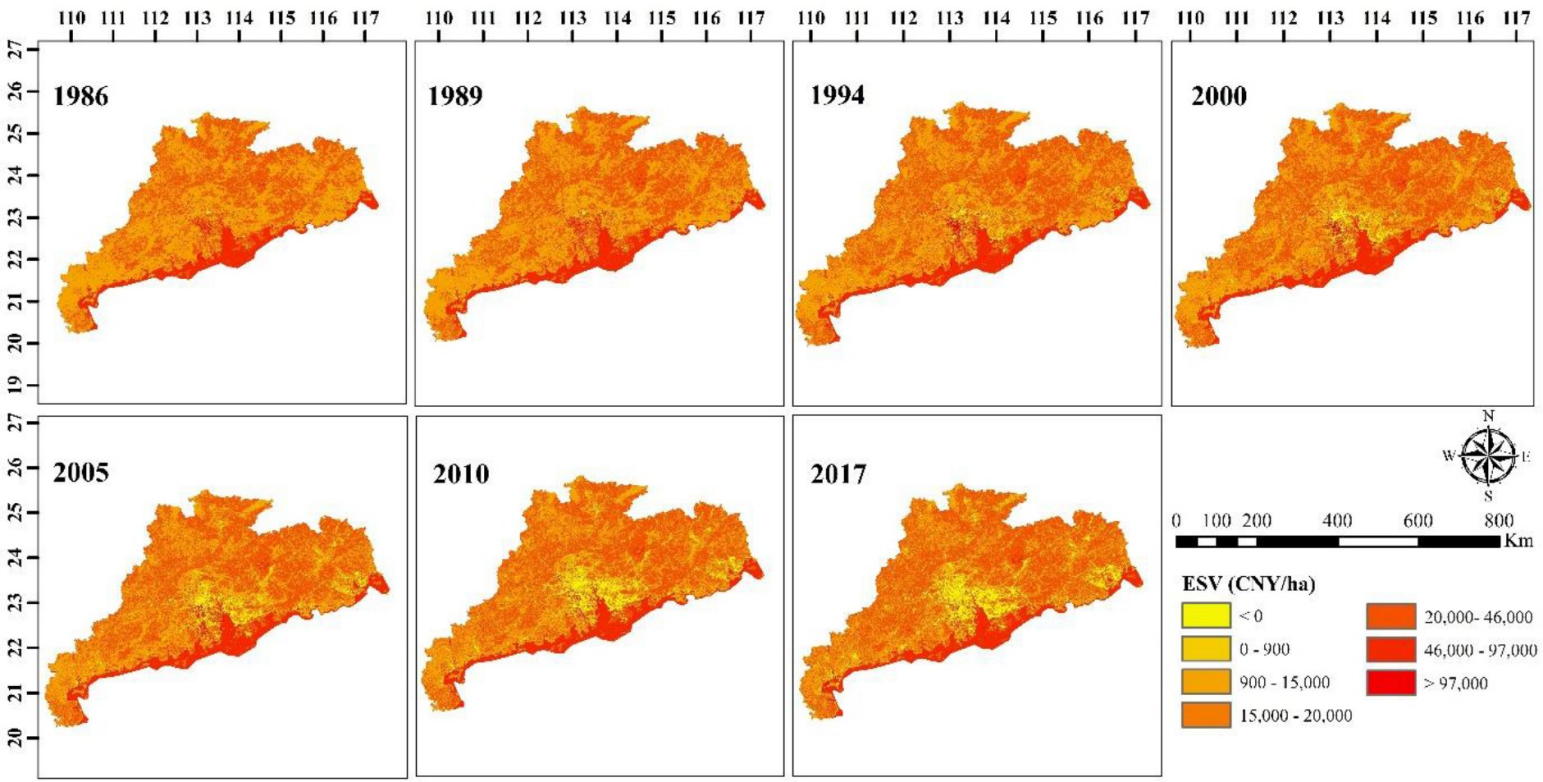
Spatially distributed total ESV in Guangdong, Hong Kong, and Macao from 1986 to 2017. The map were created using software ArcGIS 10.1 [61].

**Fig 6:**
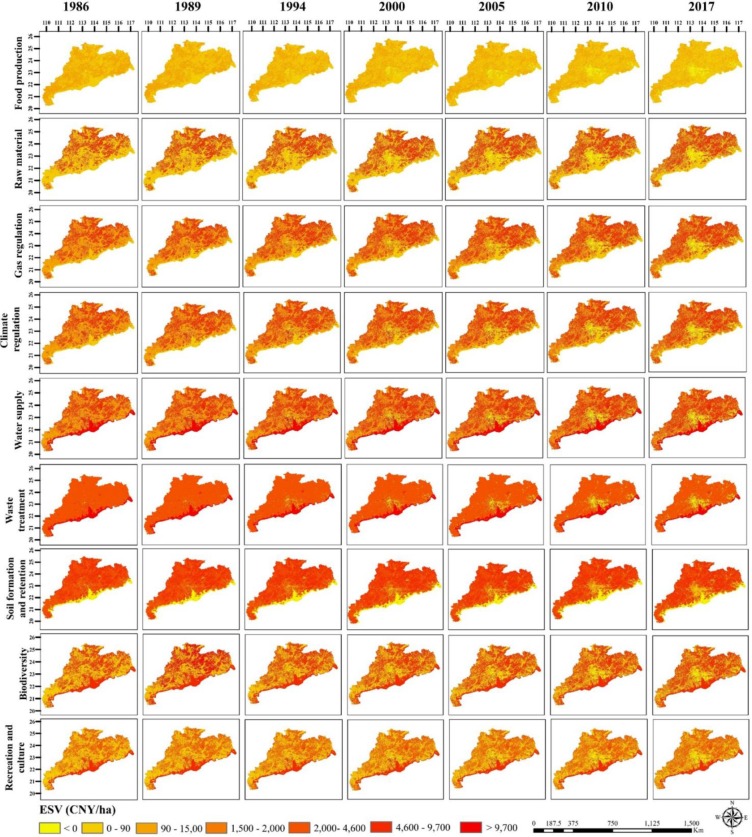
Spatial distribution of individual ecosystem functions in Guangdong, Hong Kong, and Macao from 1986 to 2017. The map were created using software ArcGIS 10.1 [61].

The ESV varied spatially across the GHKM. The ESV in the hilly and mountainous areas and in southern regions of the GHKM was greater mainly due to the forest extent. In the PRD region and on the eastern side, ESV was low because of the development of the built-up area under fast growing urbanization. The urban areas were immediately surrounded by medium value farmland and water (Figs 5 and 6). Furthermore, individual ecosystem functions such as water supply, waste treatment, climate regulation, gas regulation, and food production decreased significantly in the PRD and on the eastern side of the GHKM during the study period (Fig 6). This is mainly because unprecedented industrialization, foreign direct investment, intense human activities, and socioeconomic development have been observed in these regions. Moreover, biodiversity protection, recreation and culture, raw material, and soil formation and retention have increased during 1986 and 2017 more pronounced in the mountainous region and on the southwestern side (Fig 6). This is the result of enaction of different land policies such as the “Forestry action plan for China Agenda 21 (1995)” and “Utilization Plan (2002)”. On the other hand, they decreased in the PRD and on the eastern side (Fig 6).

### Sensitivity analysis

Sensitivity analysis was performed in order to assess the reliability of the results. The changes in the coefficient of sensitivity (CS) value must be relatively low i.e., less than one (in Eq (4)). In all cases, values of CS < 1 and often are near to zero (Table 6). This confirms that the total ESV estimation was relatively inelastic in relation to the coefficient value [16]. The CS for forest, water, farmland, and fishponds was relatively large. Forest has the highest coefficient of sensitivity, about 0.5%, due to its high coefficient value and large area. Though the water and fishponds areas were small, their CS was relatively large because of their high value coefficients. Their CS decreased from 0.29 to 0.28 and 0.05 to 0.03 during the study period (Table 6). As compared to forest and water, the CS of farmland is lower, declining from 0.22 to 0.14 during 1986–2017. The decrease in farmland and fishponds CS was mainly the result of an increase in urbanization and industrialization. Thus, in this present study, the sensitivity analysis showed that the estimation was robust despite uncertainties in the value coefficients.

**Table 6:** Percentage wise change in the coefficient of sensitivity (CS) and estimated total ESV by 50% adjustment in the value of coefficient (VC).

	1986		1989		1994		2000		2005		2010		2017	
	%	CS	%	CS	%	CS	%	CS	%	CS	%	CS	%	CS
**Forest VC±50%**	21.94	***0*.*44***	23.03	***0*.*46***	24.30	***0*.*49***	25.44	***0*.*51***	26.58	***0*.*53***	28.24	***0*.*56***	30.24	***0*.*60***
**Grassland VC±50%**	0.05	***0*.*00***	0.03	***0*.*00***	0.04	***0*.*00***	0.02	***0*.*00***	0.04	***0*.*00***	0.02	***0*.*00***	0.02	***0*.*00***
**Water VC±50%**	14.72	***0*.*29***	14.22	***0*.*28***	14.10	***0*.*28***	14.10	***0*.*28***	14.07	***0*.*28***	14.44	***0*.*29***	14.00	***0*.*28***
**Fishponds VC±50%**	2.38	***0*.*05***	2.72	***0*.*05***	3.47	***0*.*07***	3.23	***0*.*06***	3.00	***0*.*06***	2.39	***0*.*05***	1.64	***0*.*03***
**Built-up VC±50%**	-0.28	***-0*.*01***	-0.47	***-0*.*01***	-0.96	***-0*.*02***	-1.49	***-0*.*03***	-2.09	***-0*.*04***	-3.02	***-0*.*06***	-3.81	***-0*.*08***
**Bareland VC±50%**	0.00	***0*.*00***	0.00	***0*.*00***	0.00	***0*.*00***	0.00	***0*.*00***	0.01	***0*.*00***	0.00	***0*.*00***	0.00	***0*.*00***
**Farmland VC±50%**	11.18	***0*.*22***	10.47	***0*.*21***	9.05	***0*.*18***	8.69	***0*.*17***	8.39	***0*.*17***	7.93	***0*.*16***	7.03	***0*.*14***

### Patterns of economic growth and its effect on ecosystem service value

**Fig 7:**
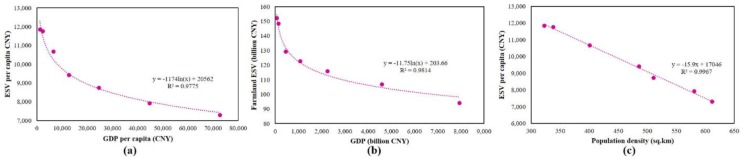
The correlation between (a) GDP per capita (CNY) and ESV per capita (CNY), (b) GDP (billion CNY) and farmland ESV (billion CNY), and (c) population density (sq.km) and ESV per capita (CNY) over the study period q986–2017).

**Fig 8:**
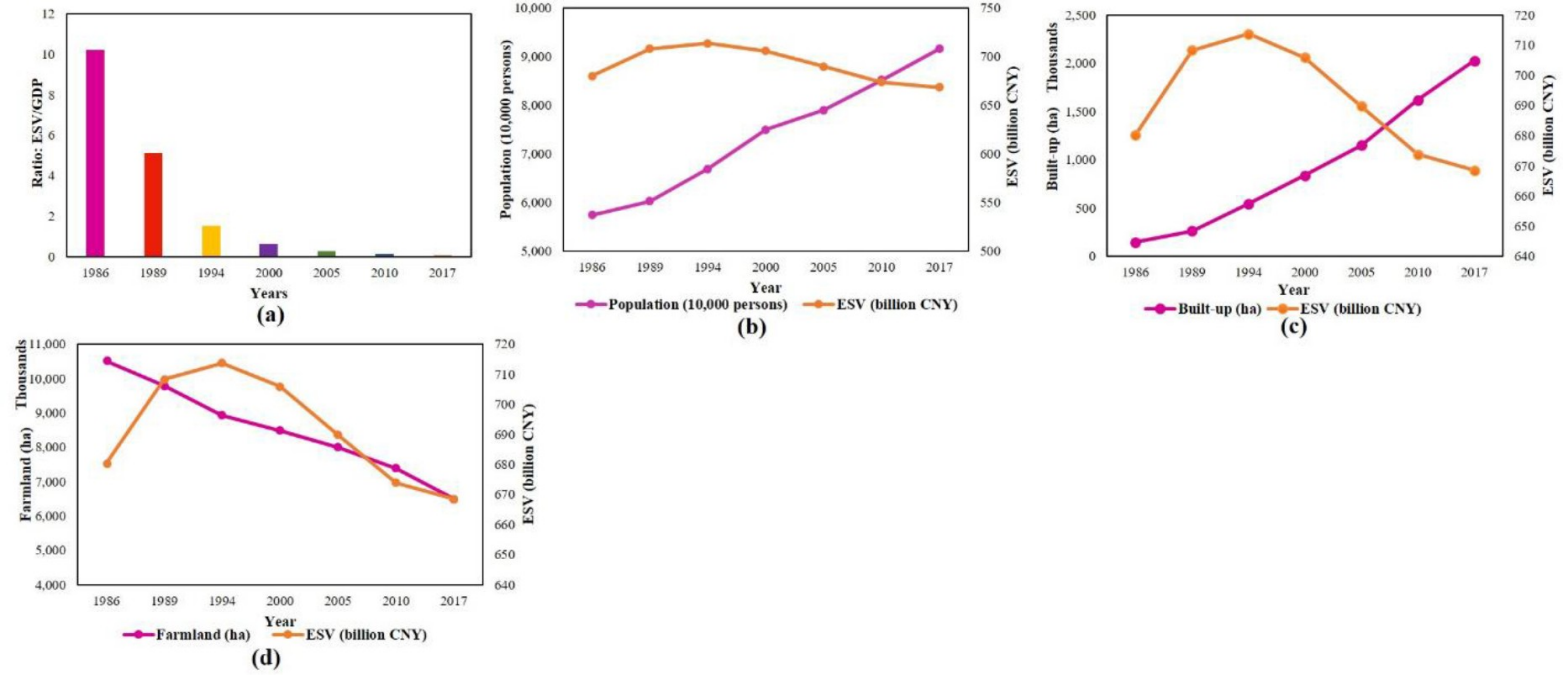
Relationship between (a) changes in the ratio of total ESV and GDP from 1986 to 2017, (b) total population (10,000 persons) and ESV (billion CNY), (c) built-up (ha) and ESV (billion CNY), and (d) farmland (ha) and ESV (billion CNY) over the study period (1986–2017).

With the increase in GDP, the accomplishment in local economic development can be assessed. In the study period, GDP increased by a factor of 119.11 times from 66.75 billion CNY in 1986 to 7951.21 billion CNY in 2017, with a yearly average growth rate of 16.67%. At the same time, ESV per capita decreased by 38.45% from 11849.22 CNY in 1986 to 7293.62 CNY in 2017. Fig 7A shows a negative non-linear relationship between GDP per capita and ESV per capita with a coefficient of determination *R*^*2*^ = 0.97. Fig 7B, a nonlinear regression analysis, demonstrated that there exists a significant negative correlation between farmland’s ESV and the GDP with a coefficient of determination *R*^*2*^ = 0.98, i.e., when GDP increased, the ESV of farmland decreased. Fig 7C indicated that the coefficient of determination between population density and ESV per capita is 0.99. Therefore, economic development and urbanization had a significant negative impact on regional ESV. Of further interest, Fig 8A shows a decline in the ratio of total ESV to total GDP during the study period. Fig 8B and 8C show that with the increase in population and built-up area ESV decreases, whereas Fig 8D reflects that decrease in farmland has a negative impact on ESV i.e., ESV decreases.

In summary, the main reason for the decrease in total ESV is the process of rapid urbanization at the expense of loss of farmland.

## Discussion

We have computed the LULC changes from 1986 to 2017 and their impact on the ESV, in the rapidly developing GHKM region. Changes in LULC and massive expansion of the built-up area has largely occupied the farmland and other natural and semi natural land cover. This has resulted in a substantial loss of ESV in certain zones while huge gains in others, with a net decrease of 1.73%. Rapid urbanization processes and industrialization have converted farmland to built-up areas. During the study period, farmland has been significantly decreased, including the conversion of farmland to built-up areas and forest. Forest and water provided the highest ESV, including water supply, waste treatment, soil formation and retention, biodiversity protection, and climate regulation. Thus, water supply, waste treatment, and food production ecosystem services faced the largest loss, while soil formation and retention and culture have achieved the greatest gain. This is because of the gain in new industrial population and to meet the needs and aspiration aligned to those new industries.

### Driving forces for land use land cover changes and ecosystem service value

After the implementation of the economic reform policy in China, GHKM region has advanced the furthest, practiced the largest socioeconomic development and population growth [20,57]. This has increased pressure to the ecology and environment and brought adverse effects on regional total ESV [15,29]. This has created numerous fascinating issues and challenges for researchers and policy and decision makers [29,69,70]. Changes in the extent and composition of the forest, grassland, fishponds, and other ecosystems have large effects on the biophysical conditions, which further influence the provision of ES and biodiversity conversion [5]. Fishponds and farmland both give various ES, for example, waste treatment, climate regulation, and biodiversity protection decrease during the study period. Both of them have greater economic benefits; they are being utilized for the construction purposes that further provoke the transformation of land use. Along with the decrease in area, the high value of water supply and waste treatment coefficients that are related with water and farmland (Table 2) have resulted in a high ESV from this land cover. The changes in LULC also influences the water supply ecosystems by shifting the transpiration, interception, and evaporation. These factors tend to increase with the increase in forest cover [5]. Forest increases with highest ESV per unit area propelled by local government after implementing “Greener Guangdong” policy promoting the construction of forest protection system. This has encouraged farmers to establish horticultural plantations and forest industry development in the GHKM, especially since 1990 [71,72].

In the process of urban expansion and industrialization, rural settlement and agricultural land depletion have experienced significant loss, which has a substantial negative effect on ESV and food security. At the end of 2013, the government established a program, namely “Farmland Protection Red Line 0.12 billion hectares (1.8 billion mu)” with the aim to maintain 1.8 billion mu farmland. Under the current scenario of rapid urbanization process, it would be very difficult to keep a target of 0.12 billion of farmland in the future [17,73]. Therefore, farmland protection as well as fishponds, both need to be considered on a first priority.

The results of this study show consistency with past literature regarding the effect of LULC changes and urbanization on the ESV at a variable rate, ranging from significant decreases to a modest increase in service value, with the majority report a modest decrease in service value. Moreover, economic growth seems to be in conflict with ecological protection as this study also shows that ESV and ESV per capita decreased significantly with the continuous increase in total GDP and GDP per capita over the past three decades in the GHKM. The main reason for such a decrease in ESV is the transformation of natural and semi natural resources into built-up land [4,6,10,25,29,41], typically resulting in lower or negative values of services. Nonetheless, even in PRD, a fast urbanizing GHKM region, urban expansion is only one of the various LULC change happening concurrently. A range of other LULC changes corresponding with the increase of built-up area also took place. Such changes include a transformation of farmland to forest, a high service value land use. To some extent, this transformation negates the adverse effect of urban expansion on ESV in the GHKM [20].

### Implication for planning sustainable development

The study presented in this paper clearly demonstrates the net decline in the ESV supply i.e., -1.73. Therefore, GHKM needs improved planning regarding sustainability of ecosystems and smart land use. Such planning should involve environmental, economic, and social considerations in order that the sustainability of services antagonistically influenced by fast urban expansion, for example, gas regulation, water supply, waste treatment, climate regulation, and food production must be stressed for improvement. Therefore, planning and decisions should focus on protecting farmland and fishponds to reverse the unsustainable deterioration in these ecosystem services. Similarly, the protection of forest and water is also important because they also comprise of high ecosystem services value. This could be accomplished through planning protocols and setting the sustainability targets for local ecosystem services by using different decision analysis methods such as triage planning [74] and spatial optimization algorithms [20,75]. This could reduce the future hazard for ESV. In summary, ESV has the great potential to inform policy and decision makers by highlighting the advantages of sustainable ecosystem management.

### Limitations

In this study, the method used to calculate the ESV was proposed by Costanza et al. (1997a,b), and adjusted by Xie et al. (2003) according to the Chinese terrestrial terrain. The ESV was then derived by multiplying each land use class with a corresponding ecosystem coefficient value. Although, estimated results produced by this method have been criticized because of used at coarse resolution, uncertainties due to complex, dynamic, and nonlinear nature of ecosystems [76,77], limiting economic valuation, and double scale problems [25,41,77,78].

The biomes used as a proxy for LULC classes but does not match precisely in each case [36]. Additionally, heterogeneity in an ecosystem made the accuracy of the adjusted coefficient values in doubt [41]. Although, a diverse range of valuation methods are available but, each and every method may prompt “refer” to different estimated values, hence causing a criticism in the ecosystem service valuation method. Thus it is essential to realize that the precise evaluation of the coefficients for time series analysis is less critical than the cross-sectional analysis. This is because the coefficients will, in general, have less influence regarding the estimation of directional change than that of the magnitude of ecosystem values [25,41]. In this study, the supposition that coefficient of ESV remains constant over time, allows a comparison of minimal change with time. However, in reality, it is unlikely that values remain constant [79]. This study attempted to adjust the value of coefficients on the basis of study area data, but still it remains a general estimation and unable to capture the spatial heterogeneity among the supply of ecosystem services within the LULC classes [20,41]. This method, however, will remain a convenient mode to integrate the effect of LULC changes across numerous ecosystem services and also identify minimal change with time in the provision of ecosystem services. Moreover, sensitivity analysis demonstrates that total ESV estimated in this study were relatively inelastic with respect to the value coefficient and despite of uncertainties our estimation up to some extent is robust.

The reliability of a proxy based method can be increased by using remotely sensed high resolution images in combination with field survey. The field survey can empower LULC mapping at high accuracy. The methods used in this study also suppose that the value of each ecosystem service, given by each LULC over the study area is homogeneous, as the value coefficients are regionally downscaled values. Instead, in reality, values change spatially. This is a drawback of methods which can be overcome by incorporating biophysical and economic systems spatial models [51] and by doing field survey for higher scale economic valuation of the supply of ecosystem services on local level [20,80,81].

## Conclusions

This study has revealed the impact of LULC changes on ESV resulting from urban expansion, industrialization, and socioeconomic development in the GHKM between 1986 and 2017. The changes in the ESV show a close relationship with socioeconomic growth in the study area. The result showed that the built-up area had expanded by 1260.02% over the last three decades, with an average annual growth rate of 8.41%, produced mainly at the expense of the reduction of farmland, together with other concurrent non-urban LULC changes. This has placed strong pressure on both natural and semi-natural ecosystems.

The total ESV decreased by 1.73% (11.77 billion CNY) between 1986 and 2017. This decrease in the value of ecosystem services is associated with a decrease in the total area of farmland, fishponds, and water. This also signifies the dynamics and complexity of the individual ESV as notably some services value decreased significantly while others increased substantially. Forest generated the highest percentage of the total ESV (approximately 50%) and together with fishponds, water, and farmland produced more than 90% of the total ESV, showing that these four LULC classes have an important role in supplying ecosystem services.

Regarding the total ESV, the highest contribution is made by water supply followed by waste treatment ecological function. Their contribution represents approximately 45% of the total. The result shows that there exists a substantial negative correlation between farmland ESV and the GDP. The ESV for farmland was higher in 1986, but tended to decrease rapidly during the study period as a consequence of the burgeoning industrialization and development. In regional land use planning and decision analysis, priority must be given to those services which can contribute to the sustainability of everyday life, particularly which can be adversely influenced by urban expansion such as water supply, gas regulation, climate regulation, and food production. Therefore, the fragile ecological environment in the GHKM clearly indicate that stakeholders and planners need to highlight the protection of such as farmland and fishponds to achieve the sustainable utilization of land resources and organized economic and environmental development.

Furthermore, by using remote sensing data, the land cover class can be utilized as a proxy for ecosystem services, with corresponding land cover classes equal to biomes, thus, making the ecosystem valuation possible for larger regions. Further research should expand or design such methods that can more precisely evaluate these coefficients for the authenticity of the resulting estimate reliant upon the precision of the coefficient value.

## Supporting information

S1 Table Land use transitions in Guangdong, Hong Kong, and Macao between 1986 and 2017(km^2^). (DOCX)

S2 Table Value of ecosystem services function from 1986 to 2017. (DOCX)

## References

[1] Daily GC. Nature’s Services: Societal Dependence on Natural Ecosystems [Internet]. Island Press, Washington, DC; 1997 Available from: http://doi.wiley.com/10.1111/j.1469-8749.2012.04283.x

[2] Costanza R, D’Arge R, de Groot R, Farber S, Grasso M, Hannon B, et al The value of the world’s ecosystem services and natural capital. Nature [Internet]. 1997 5;387(6630):253–60. Available from: http://www.esd.ornl.gov/benefits_conference/nature_paper.pdf%5Cnpapers3://publication/uuid/9E5D4B1B-DA99-4FDE-95A2-E07C92068349

[3] Costanza R, Batabyal AA, Cumberland J, Daly H, Goodland R, Norgaard R. An Introduction to Ecological Economics [Internet]. St Lucie Press, FL, USA; 1997 Available from: https://www.jstor.org/stable/4003165?origin=crossref

[4] Feng XY, Luo GP, Li CF, Dai L, Lu L. Dynamics of ecosystem service value caused by land use changes in manas river of Xinjiang, China. Int J Environ Res. 2012;6(2):499–508.

[5] Zhan J. Impacts of Land-use Change on Ecosystem Services [Internet]. Zhan J, editor. Springer Heidelberg New York Dordrecht London; 2015 Available from: http://www.springer.com/series/10180

[6] Lin X, Xu M, Cao C, Singh RP, Chen W, Ju H. Land-use/land-cover changes and their influence on the ecosystem in Chengdu City, China during the period of 1992–2018. Sustain. 2018;10(10):1–20.

[7] Zhang Y, Zhao L, Liu J, Liu Y, Li C. The Impact of Land Cover Change on Ecosystem Service Values in Urban Agglomerations along the Coast of the Bohai Rim, China. Sustainability [Internet]. 2015 8 5;7(8):10365–87. Available from: http://www.mdpi.com/2071-1050/7/8/10365

[8] Łowicki D, Walz U. Gradient of Land Cover and Ecosystem Service Supply Capacities- A Comparison of Suburban and Rural Fringes of Towns Dresden (Germany) and Poznan (Poland). Procedia Earth Planet Sci [Internet]. 2015;15:495–501. Available from: 10.1016/j.proeps.2015.08.057

[9] Zorrilla-Miras P, Palomo I, Gómez-Baggethun E, Martín-López B, Lomas PL, Montes C. Effects of land-use change on wetland ecosystem services: A case study in the Doñana marshes (SW Spain). Landsc Urban Plan [Internet]. 2014;122:160–74. Available from: 10.1016/j.landurbplan.2013.09.013

[10] Ye Y, Zhang J, Bryan BA, Gao L, Qin Z, Chen L, et al Impacts of Rapid Urbanization on Ecosystem Services along Urban-Rural Gradients: A Case Study of the Guangzhou-Foshan Metropolitan Area, South China. Ecoscience [Internet]. 2018;25(3):235–47. Available from: 10.1080/11956860.2018.1442086

[11] Costanza R, de Groot R, Sutton P, van der Ploeg S, Anderson SJ, Kubiszewski I, et al Changes in the global value of ecosystem services. Glob Environ Chang [Internet]. 2014 5;26(1):152–8. Available from: 10.1016/j.gloenvcha.2014.04.002

[12] Fu B, Zhang L, Xu Z, Zhao Y, Wei Y, Skinner D. Ecosystem services in changing land use. J Soils Sediments [Internet]. 2015 4 13;15(4):833–43. Available from: http://link.springer.com/10.1007/s11368-015-1082-x

[13] Gaglio M, Aschonitis VG, Gissi E, Castaldelli G, Fano EA. Land use change effects on ecosystem services of river deltas and coastal wetlands: case study in Volano–Mesola–Goro in Po river delta (Italy). Wetl Ecol Manag [Internet]. 2017 2;25(1):67–86. Available from: http://link.springer.com/10.1007/s11273-016-9503-1

[14] Li J, Wang W, Hu G, Wei Z. Changes in ecosystem service values in Zoige Plateau, China. Agric Ecosyst Environ [Internet]. 2010;139(4):766–70. Available from: 10.1016/j.agee.2010.10.019

[15] Yirsaw E, Wu W, Temesgen H, Bekele B. Effect of temporal land use/land cover changes on ecosystem services value in coastal area of China: The case of Su-Xi-Chang region. Appl Ecol Environ Res. 2016;14(3):409–22.

[16] Mamat A, Halik Ü, Rouzi A. Variations of ecosystem service value in response to land-use change in the Kashgar Region, Northwest China. Sustain. 2018;10(1).

[17] Chen J, Guo F, Wang H, Wang Z, Wu Y. Urban Land Revenue and Sustainable Urbanization in China: Issues and Challenges. Sustainability [Internet]. 2018 6 21;10(7):2111 Available from: http://www.mdpi.com/2071-1050/10/7/2111

[18] Song W, Deng X. Land-use/land-cover change and ecosystem service provision in China. Sci Total Environ [Internet]. 2017;576:705–19. Available from: 10.1016/j.scitotenv.2016.07.078 27810757

[19] United Nations Department of Economic and Social Affairs Population Division. World Urbanization Prospects: The 2017 Revision. 2017.

[20] Ye Y, Bryan BA, Zhang J, Connor JD, Chen L, Qin Z, et al Changes in land-use and ecosystem services in the Guangzhou-Foshan Metropolitan Area, China from 1990 to 2010: Implications for sustainability under rapid urbanization. Ecol Indic [Internet]. 2018 10;93(August 2017):930–41. Available from: https://linkinghub.elsevier.com/retrieve/pii/S1470160X18303728

[21] Elmqvist T, Setälä H, Handel S, van der Ploeg S, Aronson J, Blignaut J, et al Benefits of restoring ecosystem services in urban areas. Curr Opin Environ Sustain [Internet]. 2015 6;14:101–8. Available from: https://linkinghub.elsevier.com/retrieve/pii/S1877343515000433

[22] Gómez-Baggethun E, Barton DN. Classifying and valuing ecosystem services for urban planning. Ecol Econ [Internet]. 2013 2;86:235–45. Available from: 10.1016/j.ecolecon.2012.08.019

[23] Yan-qiong Y, Jia-en Z, LI Yun LY. Impact of Socio-economic Driving Factors of Agricultural Land Use Change on Agroecosystem Service Values in Guangdong Province. Res Agric Mod. 2011;32(6).

[24] Chang-ping Z. Assessment on the Eco-enviornment and the Land Use Based on the Ecosystem Service Value—A Case of Guangdong Province, China. Asian Agric Res. 2010;2(4):34–36,40.

[25] Liu Y, Li J, Zhang H. An ecosystem service valuation of land use change in Taiyuan City, China. Ecol Modell [Internet]. 2012;225:127–32. Available from: 10.1016/j.ecolmodel.2011.11.017

[26] Wang Z, Zhang B, Zhang S, Li X, Liu D, Song K, et al Changes of Land Use and of Ecosystem Service Values in Sanjiang Plain, Northeast China. Environ Monit Assess [Internet]. 2006 1;112(1–3):69–91. Available from: 10.1007/s10661-006-0312-5 16404535

[27] Hao F, Lai X, Ouyang W, Xu Y, Wei X, Song K. Effects of Land Use Changes on the Ecosystem Service Values of a Reclamation Farm in Northeast China. Environ Manage [Internet]. 2012 11 9;50(5):888–99. Available from: 10.1007/s00267-012-9923-5 22961612

[28] Hu X, Wu C, Hong W, Qiu R, Qi X. Impact of land-use change on ecosystem service values and their effects under different intervention scenarios in Fuzhou City, China. Geosci J [Internet]. 2013 12 13;17(4):497–504. Available from: http://link.springer.com/10.1007/s12303-013-0040-0

[29] Wu K, Ye X, Qi Z, Zhang H. Impacts of land use/land cover change and socioeconomic development on regional ecosystem services: The case of fast-growing Hangzhou metropolitan area, China. Cities [Internet]. 2013 4;31:276–84. Available from: 10.1016/j.cities.2012.10.011

[30] Li F, Ye YP, Song BW, Wang RS, Tao Y. Assessing the changes in land use and ecosystem services in Changzhou municipality, Peoples’ Republic of China, 1991–2006. Ecol Indic [Internet]. 2014 7;42:95–103. Available from: https://linkinghub.elsevier.com/retrieve/pii/S1470160X13004305

[31] Chen J, Sun B-M, Chen D, Wu X, Guo L-Z, Wang G. Land Use Changes and Their Effects on the Value of Ecosystem Services in the Small Sanjiang Plain in China. Sci World J [Internet]. 2014;2014:1–7. Available from: http://www.hindawi.com/journals/tswj/2014/752846/10.1155/2014/752846 PMC3972864 24741356

[32] Cai Y Bin, Li HM, Ye XY, Zhang H. Analyzing three-decadal patterns of land use/land cover change and regional ecosystem services at the landscape level: Case study of two coastal metropolitan regions, Eastern China. Sustain. 2016;8(8):1–21.

[33] Wang M, Sun X. Potential impact of land use change on ecosystem services in China. Environ Monit Assess [Internet]. 2016;188(4). Available from: 10.1007/s10661-016-5245-z 27021691

[34] Baró F, Haase D, Gómez-Baggethun E, Frantzeskaki N. Mismatches between ecosystem services supply and demand in urban areas: A quantitative assessment in five European cities. Ecol Indic [Internet]. 2015 8;55:146–58. Available from: 10.1016/j.ecolind.2015.03.013

[35] Cai Y-B, Zhang H, Pan W-B, Chen Y-H, Wang X-R. Land use pattern, socio-economic development, and assessment of their impacts on ecosystem service value: study on natural wetlands distribution area (NWDA) in Fuzhou city, southeastern China. Environ Monit Assess [Internet]. 2013 6 10;185(6):5111–23. Available from: http://link.springer.com/10.1007/s10661-012-2929-x 23054291 10.1007/s10661-012-2929-x

[36] Kreuter UP, Harris HG, Matlock MD, Lacey RE. Change in ecosystem service values in the San Antonio area, Texas. Ecol Econ [Internet]. 2001 12;39(3):333–46. Available from: http://linkinghub.elsevier.com/retrieve/pii/S0921800901002506

[37] Ye Y, Zhang J, Chen L, Ouyang Yi, Parajuli P. Dynamics of ecosystem service values in response to landscape pattern changes from 1995 to 2005 in Guangzhou, Southern China. Appl Ecol Environ Res. 2015;13(1):85–97.

[38] Mendoza-González G, Martínez ML, Lithgow D, Pérez-Maqueo O, Simonin P. Land use change and its effects on the value of ecosystem services along the coast of the Gulf of Mexico. Ecol Econ [Internet]. 2012 10;82:23–32. Available from: 10.1016/j.ecolecon.2012.07.018

[39] Estoque RC, Murayama Y. Landscape pattern and ecosystem service value changes: Implications for environmental sustainability planning for the rapidly urbanizing summer capital of the Philippines. Landsc Urban Plan [Internet]. 2013 8;116:60–72. Available from: 10.1016/j.landurbplan.2013.04.008

[40] TEEB. Urbanization, Biodiversity and Ecosystem Services: Challenges and Opportunities A Global Assessment. In: Elmqvist T, Fragkias M, Goodness J, Güneralp B, Marcotullio PJ, McDonald RI, et al, editors. Dordrecht: Springer Netherlands; 2013 p. 57–63. Available from: http://link.springer.com/10.1007/978-94-007-7088-1_5

[41] Tianhong L, Wenkai L, Zhenghan Q. Variations in ecosystem service value in response to land use changes in Shenzhen. Ecol Econ [Internet]. 2010 5;69(7):1427–35. Available from: 10.1016/j.ecolecon.2008.05.018

[42] Long H, Liu Y, Hou X, Li T, Li Y. Effects of land use transitions due to rapid urbanization on ecosystem services: Implications for urban planning in the new developing area of China. Habitat Int [Internet]. 2014 10;44:536–44. Available from: 10.1016/j.habitatint.2014.10.011

[43] Su S, Li D, Hu Y, Xiao R, Zhang Y. Spatially non-stationary response of ecosystem service value changes to urbanization in Shanghai, China. Ecol Indic. 2014;45:332–9.

[44] Sudhira HS, Nagendra H. Local Assessment of Bangalore: Graying and Greening in Bangalore–Impacts of Urbanization on Ecosystems, Ecosystem Services and Biodiversity In: Elmqvist T, Fragkias M, Goodness J, Güneralp B, Marcotullio PJ, McDonald RI, et al, editors. Urbanization, Biodiversity and Ecosystem Services: Challenges and Opportunities [Internet]. Dordrecht: Springer Netherlands; 2013 p. 75–91. Available from: 10.1007/978-94-007-7088-1_7

[45] Yi H, Güneralp B, Filippi AM, Kreuter UP, Güneralp İ. Impacts of Land Change on Ecosystem Services in the San Antonio River Basin, Texas, from 1984 to 2010. Ecol Econ [Internet]. 2017 5;135:125–35. Available from: https://linkinghub.elsevier.com/retrieve/pii/S0921800916306899

[46] Han Z, Song W, Deng X. Responses of ecosystem service to land use change in Qinghai Province. Energies. 2016;9(4).

[47] Wang Y, Gao J, Wang J, Qiu J. Value assessment of ecosystem services in nature reserves in Ningxia, China: A response to ecological restoration. PLoS One. 2014;9(2). 10.1371/journal.pone.0089174 PMC3929645 24586571

[48] Li T, Ding Y. Spatial disparity dynamics of ecosystem service values and GDP in Shaanxi Province, China in the last 30 years. Hewitt J, editor. PLoS One [Internet]. 2017 3 30;12(3):e0174562 Available from: 10.1371/journal.pone.0174562 28358918 PMC5373591

[49] Fang X, Tang G, Li B, Han R. Spatial and Temporal Variations of Ecosystem Service Values in Relation to Land Use Pattern in the Loess Plateau of China at Town Scale. Machado RB, editor. PLoS One [Internet]. 2014 10 20;9(10):e110745 Available from: 10.1371/journal.pone.0110745 25329311 PMC4203848

[50] Xia T, Wu W, Zhou Q, Tan W, Verburg PH, Yang P, et al Modeling the spatio-temporal changes in land uses and its impacts on ecosystem services in Northeast China over 2000–2050. J Geogr Sci [Internet]. 2018 11 28;28(11):1611–25. Available from: http://link.springer.com/10.1007/s11442-018-1532-7

[51] Bryan BA, Crossman ND. Impact of multiple interacting financial incentives on land use change and the supply of ecosystem services. Ecosyst Serv [Internet]. 2013 6;4:60–72. Available from: 10.1016/j.ecoser.2013.03.004

[52] Bryan BA, Crossman ND, Nolan M, Li J, Navarro J, Connor JD. Land use efficiency: anticipating future demand for land-sector greenhouse gas emissions abatement and managing trade-offs with agriculture, water, and biodiversity. Glob Chang Biol [Internet]. 2015 11;21(11):4098–114. Available from: 10.1111/gcb.13020 26147156

[53] Liu J, Dietz T, Carpenter SR, Alberti M, Folke C, Moran E, et al Complexity of Coupled Human and Natural Systems. Science (80-) [Internet]. 2007 9 14;317(5844):1513–6. Available from: http://www.sciencemag.org/cgi/doi/10.1126/science.1144004 17872436 10.1126/science.1144004

[54] Wang Z, Wang Z, Zhang B, Lu C, Ren C. Impact of land use/land cover changes on ecosystem services in the Nenjiang River Basin, Northeast China. Ecol Process [Internet]. 2015 12 30;4(1):11 Available from: 10.1186/s13717-015-0036-y

[55] Zhou J, Sun L, Zang SY, Wang K, Zhao JY, Li ZX, et al Effects of the land use change on ecosystem service value. Glob J Environ Sci Manag. 2017;3(2):121–30.

[56] Shobairi SO, Li M. Dynamic Modelling of VFC from 2000 to 2010 Using NDVI and DMSP/OLS Time Series: A Study in Guangdong Province, China. J Geogr Inf Syst [Internet]. 2016;8(2):205–23. Available from: http://www.scirp.org/journal/PaperDownload.aspx?DOI=10.4236/jgis.2016.82019

[57] Hasan S, Shi W, Zhu X, Abbas S. Monitoring of Land Use/Land Cover and Socioeconomic Changes in South China over the Last Three Decades Using Landsat and Nighttime Light Data. Remote Sens [Internet]. 2019 7 11;11(14):1658 Available from: https://www.mdpi.com/2072-4292/11/14/1658

[58] Li C, Kuang Y, Huang N, Zhang C. The long-term relationship between population growth and vegetation cover: An empirical analysis based on the panel data of 21 cities in Guangdong province, China. Int J Environ Res Public Health. 2013;10(2):660–77. 10.3390/ijerph10020660 23435589 PMC3635169

[59] Li L, Wang Y. Land Use / Cover Change from 2001 to 2010 and its Socioeconomic Determinants in Guangdong Province, A Rapid Urbanization Area of China. J Agric Sci. 2015;86(20).

[60] Xiowei X, Xiangxin C, Jianfu Y. Guangdong Statistics Yearbook 2017. In China Statistics Press; 2017.

[61] ESRI. ArcGIS Desktop: Release 10. Redlands, CA: Environmental Systems Research Institute.; 2011.

[62] Li G, Fang C, Wang S. Exploring spatiotemporal changes in ecosystem-service values and hotspots in China. Sci Total Environ [Internet]. 2016 3;545–546:609–20. Available from: 10.1016/j.scitotenv.2015.12.067 26760280

[63] XIE G, Chun-xia L, Yun-fa L, Du Z, Shuang-cheng L. Ecological assets valuation of the Tibetan Plateau. J Nat Resour. 2003;18:189–96.

[64] Dong J., Shu T., Xie H, Bao C. Calculative method for ecosystem services values of urban constructive lands and its application. J Tongji Univ (Nat Sci). 2007;35:636–40.

[65] Deng S. Dynamic effect on ecosystem services value change with regional land use change [dissertation]. Hangzhou: Zhejiang University; 2012.

[66] Zhang P, He L, Fan X, Huo P, Liu Y, Zhang T, et al Ecosystem Service Value Assessment and Contribution Factor Analysis of Land Use Change in Miyun County, China. Sustainability [Internet]. 2015 6 9;7(6):7333–56. Available from: http://www.mdpi.com/2071-1050/7/6/7333/

[67] Zhu R, Zhang Z, Wan L, Gong J, Li Y. Temporal and Spatial Variation of Ecosystem Service Value and Land Use in Dongjiang River Basin—A Case of Dongyuan County. Prog Eng Sci. 2017;42(3).

[68] Aschonitis VG, Gaglio M, Castaldelli G, Fano EA. Criticism on elasticity-sensitivity coefficient for assessing the robustness and sensitivity of ecosystem services values. Ecosyst Serv [Internet]. 2016;20:66–8. Available from: 10.1016/j.ecoser.2016.07.004

[69] Liu Y, Song Y, Arp HP. Examination of the relationship between urban form and urban eco-efficiency in china. Habitat Int [Internet]. 2012 1;36(1):171–7. Available from: 10.1016/j.habitatint.2011.08.001

[70] Wu F, Yeh AG-O. Changing Spatial Distribution and Determinants of Land Development in Chinese Cities in the Transition from a Centrally Planned Economy to a Socialist Market Economy: A Case Study of Guangzhou. Urban Stud [Internet]. 1997 11 2;34(11):1851–79. Available from: http://journals.sagepub.com/doi/10.1080/0042098975286

[71] Bui T., Yang D., Jones W., Li J. China’s Economic Powerhouse: Economic Reform in Guangdong Province. Palgrave Macmillan, New York; 2003.

[72] Chokkalingam U, Zhou Z, Toma T. Learning lessons from China ‘ s forest rehabilitation efforts National level review and special focus on Guangdong Province. Center for International Forestry Research; 2006.

[73] Tan Y, He J, Yue W, Zhang L, Wang Q. Spatial pattern change of the cultivated land before and after the second national land survey in China. J Nat Resour. 2017;32(186–197).

[74] Pendleton L, Mongruel R, Beaumont N, Hooper T, Charles M. A triage approach to improve the relevance of marine ecosystem services assessments. Mar Ecol Prog Ser. 2015;530(June):183–93.

[75] Chuai X, Huang X, Wu C, Li J, Lu Q, Qi X, et al Land use and ecosystems services value changes and ecological land management in coastal Jiangsu, China. Habitat Int [Internet]. 2016;57:164–74. Available from: 10.1016/j.habitatint.2016.07.004

[76] Limburg KE, O’Neill R V., Costanza R, Farber S. Complex systems and valuation. Ecol Econ [Internet]. 2002 6;41(3):409–20. Available from: https://linkinghub.elsevier.com/retrieve/pii/S0921800902000903

[77] Turner RK, Paavola J, Cooper P, Farber S, Jessamy V, Georgiou S. Valuing nature: Lessons learned and future research directions. Ecol Econ. 2003;46(3):493–510.

[78] Konarska KM, Sutton PC, Castellon M. The Dynamics and Value of Ecosystem Services: Integrating Economic and Ecological Perspectives: Evaluating scale dependence of ecosystem service valuation: a comparison of NOAA-AVHRR and Landsat TM datasets. Ecol Econ [Internet]. 2002;41:491–507. Available from: http://proxyiub.uits.iu.edu/login?url = http://search.ebscohost.com/login.aspx?direct=true&db=edselp&AN=S0921800902000964&site=eds-live&scope=site

[79] Zank B, Bagstad KJ, Voigt B, Villa F. Modeling the effects of urban expansion on natural capital stocks and ecosystem service flows: A case study in the Puget Sound, Washington, USA. Landsc Urban Plan [Internet]. 2016;149:31–42. Available from: 10.1016/j.landurbplan.2016.01.004

[80] Bryan BA, Grandgirard A, Ward JR. Quantifying and Exploring Strategic Regional Priorities for Managing Natural Capital and Ecosystem Services Given Multiple Stakeholder Perspectives. Ecosystems [Internet]. 2010 6 25;13(4):539–55. Available from: http://link.springer.com/10.1007/s10021-010-9339-0

[81] Raymond CM, Bryan BA, MacDonald DH, Cast A, Strathearn S, Grandgirard A, et al Mapping community values for natural capital and ecosystem services. Ecol Econ [Internet]. 2009;68(5):1301–15. Available from: 10.1016/j.ecolecon.2008.12.006

