# Supplementary material for: Impact of land use land cover changes on ecosystem service value – A case study of Guangdong, Hong Kong, and Macao in South China
Source: PLoS One. 2020 Apr 8;15(4):e0231259. doi: 10.1371/journal.pone.0231259 (PMC7141676; doi:10.1371/journal.pone.0231259)
Supplement: S1 Table — (DOCX) [file pone.0231259.s001.docx]

**Table S1.** Land use transitions in Guangdong, Hong Kong, and Macao between 1986 and 2017(km^2^).

|  | | **2017** | | | | | | |  |
| --- | --- | --- | --- | --- | --- | --- | --- | --- | --- |
|  | **Land use type** | **Forest** | **Grassland** | **Water** | **Fishponds** | **Built-up** | **Bareland** | **Farmland** | **Total/LSE^1^** |
| **1986** | **Forest** | 62,031.53 | 60.13 | 211.53 | 310.49 | 2586.82 | 57.05 | 0.00 | 65,257.55 |
|  | **Grassland** | 285.24 | 63.82 | 2.69 | 2.30 | 80.75 | 2.37 | 22.93 | 460.11 |
|  | **Water** | 167.71 | 14.26 | 19,268.44 | 420.14 | 838.32 | 9.89 | 76.72 | 20,795.47 |
|  | **Fishponds** | 299.74 | 8.88 | 532.01 | 456.97 | 1074.51 | 5.75 | 85.50 | 2463.35 |
|  | **Built-up** | 0.00 | 0.00 | 0.00 | 0.00 | 1488.35 | 0.00 | 0.00 | 1488.35 |
|  | **Bareland** | 149.99 | 1.27 | 84.13 | 18.32 | 330.99 | 15.90 | 151.56 | 752.16 |
|  | **Farmland** | 25,449.98 | 41.35 | 544.48 | 466.39 | 13,841.81 | 184.44 | 64,595.48 | 105,123.93 |
|  | **Total/LSL^2^** | 88,384.19 | 189.72 | 20,643.28 | 1674.61 | 20,241.55 | 275.40 | 64,932.19 | 196,340.94 |
| ^1^LSE = Earlier landscape, ^2^LSL = Later landscape | | | | | | | | | |
