# Supplementary material for: Impact of land use land cover changes on ecosystem service value – A case study of Guangdong, Hong Kong, and Macao in South China
Source: PLoS One. 2020 Apr 8;15(4):e0231259. doi: 10.1371/journal.pone.0231259 (PMC7141676; doi:10.1371/journal.pone.0231259)
Supplement: S2 Table — (DOCX) [file pone.0231259.s002.docx]

**Table S2**. Value of ecosystem services function from 1986 to 2017.

| **ESV_f_ billion(CNY/Yr)** | **1986** | **1989** | **1994** | **2000** | **2005** | **2010** | **2017** | **%** | **Rank** | **Tendency** |
| --- | --- | --- | --- | --- | --- | --- | --- | --- | --- | --- |
| **Food** | 23.99 | 22.63 | 20.99 | 20.08 | 19.11 | 17.83 | 16.04 | 2.91 | 9 |  |
| **Raw material** | 37.80 | 40.95 | 43.25 | 44.61 | 45.41 | 46.92 | 49.54 | 6.39 | 8 |  |
| **Gas regulation** | 59.07 | 62.31 | 63.64 | 63.51 | 62.56 | 61.44 | 60.36 | 8.99 | 6 |  |
| **Climate regulation** | 67.38 | 71.13 | 75.07 | 74.66 | 73.53 | 71.55 | 70.07 | 10.41 | 5 |  |
| **Water supply** | 151.47 | 154.78 | 154.97 | 149.66 | 142.08 | 133.82 | 129.17 | 21.00 | 1 |  |
| **Waste treatment** | 141.96 | 142.71 | 142.69 | 138.37 | 132.45 | 126.21 | 121.19 | 19.55 | 2 |  |
| **Soil formation and retention** | 86.53 | 89.35 | 90.78 | 91.44 | 91.31 | 91.57 | 92.81 | 13.12 | 3 |  |
| **Biodiversity protection** | 72.55 | 75.93 | 78.41 | 79.45 | 79.74 | 80.79 | 83.11 | 11.39 | 4 |  |
| **Recreation and culture** | 39.47 | 41.73 | 43.88 | 44.05 | 43.65 | 43.70 | 44.57 | 6.23 | 7 |  |
| **Total** | 680.23 | 701.51 | 713.68 | 705.84 | 689.84 | 673.84 | 668.45 | 100.00 |  |  |
